# Untargeted metabolomics reveals N, N, N-trimethyl-L-alanyl-L-proline betaine (TMAP) as a novel biomarker of kidney function

**DOI:** 10.1038/s41598-019-42992-3

**Published:** 2019-05-02

**Authors:** Thomas J. Velenosi, Benjamin K. A. Thomson, Nicholas C. Tonial, Adrien A. E. RaoPeters, Megan A. Mio, Gilles A. Lajoie, Amit X. Garg, Andrew A. House, Bradley L. Urquhart

**Affiliations:** 1 Department of Physiology and Pharmacology, Schulich School of Medicine and Dentistry, The University of Western Ontario, Ontario, Canada; 2 Division of Nephrology, Department of Medicine, Schulich School of Medicine and Dentistry, The University of Western Ontario, Ontario, Canada; 3 Department of Biochemistry, Schulich School of Medicine and Dentistry, The University of Western Ontario, Ontario, Canada; 4 Lawson Health Research Institute, London, Canada; 5 Department of Epidemiology and Biostatistics, Schulich School of Medicine and Dentistry, The University of Western Ontario, Ontario, Canada

**Keywords:** Metabolomics, Chronic kidney disease

## Abstract

The diagnosis and prognosis of chronic kidney disease (CKD) currently relies on very few circulating small molecules, which can vary by factors unrelated to kidney function. In end-stage renal disease (ESRD), these same small molecules are used to determine dialysis dose and dialytic clearance. Therefore, we aimed to identify novel plasma biomarkers to estimate kidney function in CKD and dialytic clearance in ESRD. Untargeted metabolomics was performed on plasma samples from patients with a single kidney, non-dialysis CKD, ESRD and healthy controls. For ESRD patients, pre- and post-dialysis plasma samples were obtained from several dialysis modalities. Metabolomics analysis revealed over 400 significantly different features in non-dialysis CKD and ESRD plasma compared to controls while less than 35 features were significantly altered in patients with a single kidney. N,N,N-trimethyl-L-alanyl-L-proline betaine (TMAP, AUROC = 0.815) and pyrocatechol sulfate (AUROC = 0.888) outperformed creatinine (AUROC = 0.745) in accurately identifying patients with a single kidney. Several metabolites accurately predicted ESRD; however, when comparing pre-and post-hemodialysis, TMAP was the most robust biomarker of dialytic clearance for all modalities (AUROC = 0.993). This study describes TMAP as a novel potential biomarker of kidney function and dialytic clearance across several hemodialysis modalities.

## Introduction

Chronic kidney disease (CKD) is estimated to affect 11–13% of the global population^1^. CKD primarily manifests as a secondary complication of diabetes and hypertension^2,3^. Progressive renal damage is irreversible and therefore, patients with CKD must manage the disease along with associated comorbidities. Complications from comorbidities are further exacerbated by the accumulation of toxins that follows declining renal function. These complications begin when the estimated glomerular filtration rate (eGFR) declines to <60 ml/min per 1.73 m^2^ in stage 3, which represents more than half of all CKD patients^4,5^. In advanced CKD, progression to end-stage renal disease (ESRD) requires renal replacement therapy, which can include various hemodialysis (HD) and peritoneal dialysis (PD) modalities or kidney transplantation to sustain life. Patients with late-stage CKD have a 3 to 6-fold increased risk of mortality, which further increases to 8-fold after initiation of dialysis compared to age-matched subjects with normal or moderately decreased kidney function^6^. Kidney transplantation significantly decreases the risk of mortality and is the only treatment that can effectively reverse toxin accumulation^7^.

The accumulation of small molecules that are normally cleared by the kidneys is defined as uremia. The European Uremic Toxin Work Group has identified over 90 uremic metabolites^8^. Several of these uremic toxins are gut-derived and recent studies have associated gut-derived metabolites with cardiovascular events in ESRD. Indeed, indoxyl sulfate, p-cresyl sulfate and phenylacetylglutamine are associated with cardiovascular events that contribute to elevated mortality in ESRD^9–12^. Indoxyl sulfate and p-cresyl sulfate are highly protein bound metabolites that are efficiently cleared by tubular secretion in patients with functioning kidneys^13^, but undergo minimal dialytic clearance^8^. Conversely, only 20% of phenylacetylglutamine is protein-bound suggesting a higher likelihood of clearance by dialysis^14^.

Despite progress in uremic toxin identification, there is a need for new and reliable biomarkers to allow for more accurate estimation of GFR. This is especially important in children and the elderly. Biomarkers that enhance eGFR determination will help identify kidney disease patients earlier and help guide patient therapy during CKD progression, which has been recently highlighted^15^. It is well established that the use of current biomarkers including creatinine and urea has several limitations; however, the basis for treatments and diagnosis of disease rely on the measurement of these metabolites. Alternatively, the use of a metabolic fingerprint, which includes several metabolites, may provide a more robust characterization of kidney disease status. Metabolites directly reflect genetic, physiological, and environmental changes and can provide a reliable prognostic and diagnostic readout for disease progression^16^. Several metabolites can be identified and measured in various biological matrices using high-throughput metabolomics. Therefore, using metabolomics to characterize the plasma metabolic fingerprint in kidney disease can provide insight into the complications and high mortality rates that beset patients, and potentially lead to novel treatments.

To date, studies evaluating uremic metabolites have focused on CKD patients or ESRD patients undergoing conventional HD^17–19^. Therefore, there is a lack of evidence on the fate of uremic solutes and their clearance across dialysis modalities.

In this study, we aimed to identify early biomarkers of reduced kidney function by evaluating the plasma metabolic profiles of patients with a single kidney, which included living kidney donors and kidney transplant recipients, as compared to age-matched controls. Our second objective was to examine plasma metabolic perturbations in non-dialysis dependent (NDD) CKD patients and various dialysis modalities. In addition, we evaluated pre- and post-dialysis plasma to determine metabolites readily cleared by different dialysis modalities.

## Results

### Patient demographics and clinical factors

Ten subjects were recruited for each of the control, living kidney donor, kidney transplant, conventional HD and nocturnal intermittent peritoneal dialysis (NIPD) groups. There were 20 CKD patients and 5–6 patients in the short daily home hemodialysis (HHD), frequent nocturnal HHD, intermittent conventional HHD and intermittent nocturnal HHD groups (Table 1). The majority of CKD patients were in the later stages of CKD (stage 4–5 CKD; eGFR < 30 mL/min per 1.73 m^2^). All NIPD patients had residual renal function with a median residual volume of 712.5 (500–1250) mL/day. For all other dialysis dependent groups, patients were anuric except for one patient from each of the conventional HD, short daily HHD and intermittent nocturnal HHD groups.

**Table 1:** Demographics and Baseline characteristics of study population.

	Control	Living Donor	Transplant	CKD	Conventional HD	NIPD	Short Daily HHD	Intermittent Conventional HHD	Frequent Nocturnal HHD	Intermittent Nocturnal HHD
Number	10	10	10	20	10	10	6	5	5	5
Age (years)	34 (28–53)	48 (38–52)	55 (43–66)	69 (45–82)*	64 (62–77)*	65 (56–76)*	58 (52–67)	61 (45–69)	56 (40–60)	40 (30–54)
Body Mass Index (kg/m^2^)	27.1 (5.3)	25.5 (3.6)	27.2 (4.1)	25.6 (3.8)	28.9 (5.2)	26.3 (5.3)	32.8 (6)	33.7 (11)	32 (8.8)	24.8 (3.1)
Sex (M/F)	3/7	3/7	4/6	10/10	6/4	6/4	5/1	2/3	3/2	2/3
Race (C/B/A/F/O)	(10/0/0/0/0)	(9/1/0/0/0)	(10/0/0/0/0)	(19/1/0/0/0)	(10/0/0/0/0)	(8/0/1/1/0)	(6/0/0/0/0)	(4/1/0/0/0)	(5/0/0/0/0)	(4/0/0/0/1)
Etiology (DM/PG/SG/IN/H/CHC/M)^A^			(1/5/1/1/0/0/2)	(4/1/3/0/5/2/5)	(5/1/0/0/2/0/2)	(2/0/1/0/1/0/6)	(2/0/0/0/1/3/0)	(2/1/1/0/0/1/0)	(1/2/0/0/0/1/0/1)	(0/2/0/0/1/2/0)
Diabetes (N/Y)	10/0	10/0	8/2	15/5	14/6	7/3	4/2	3/2	4/1	5/0
eGFR (mL/min/1.73 m2) (RV → 1YR)^B^	93.5 (78.5–98.25) → 89 (76.75–98.75)	85.5 (69.25–91.75) → 61.5 (55.25–67.75)^†^	68.03 (56.19–74.33)	12.51 (7.85–18.86)*		6.385 (4.54–9.13)*				
Serum Cr (μmol/L) (RV → 1YR)^B^	73 (60.75–85) → 71 (65.25–90.5)	74 (67–82.5) → 99.5 (86–115)^†^	97.5 (77.75–109.5)	361 (281.3–503.8)*	560.5 (425.3–807)*	683.5 (537–969.3)*	832.5 (571.8–863.3)*	521 (431–698.5)*	596 (398.5–758.5)*	596 (495.5–758.5)*
Serum Hb (g/L) (RV → 1YR)^B^	136.4 (14.29) → 140.7 (12.6)	131.9 (11.82) → 131.8 (11.08)	133.5 (21.04)	113.9 (15.72)	104 (14.92)*	105 (11.3)*	125 (19.91)	108.8 (23.15)	105.8 (13.52)	123.8 (14.67)
HD or PD Frequency (sessions/wk)					3 (3–3)	6 (5.5–7)	6 (4.5–6)	3 (3–4)	5 (5–6)	3 (3–3.5)
HD or PD weekly duration (min/week)					630 (608–720)	2880 (2880–3068)^D^	975 (680–1125)^DE^	720 (585–840)^DE^	2400 (2130–2460)^D^	1440 (1260–1750)
Kt/V (single pooled for HD, weekly for NIPD)					1.52 (1.25–1.62)	1.9 (1.81–2.26)	1.36 (1.19–1.72)	1.52 (1.2–2.02)	2.22 (1.92–2.97)^DF^	2.15 (1.8–2.93)^D^
Hemodialysis Membrane Surface Area (m^2^)^C^					1.5 (1.5–1.5)		1.8 (1.5–2.2)	1.5 (1.5–1.8)	1.5 (1.2–1.5)	1.5 (1.5–1.8)
Hemodialysis Qb (blood flow rate) (mL/min)					350 (350–381)		400 (375–425)	400 (400–500)	300 (225–350)	300 (250–350)
Hemodialysis Qd (dialysate flow rate) (mL/min)					500 (500–500)		500 (500–550)	500 (500–750)	300 (300–400)	500 (300–500)

Demographic characteristics are presented as mean (SD) or median (interquartile range). Statistical differences were determined using the Kruskal-Wallis test followed by Dunn’s Test.

Age and body mass index for Control and Living Donor groups were obtained at recruitment visit (RV).

*p < 0.05 compared to control.

^†^p < 0.05 by paired analysis.

^A^DM = diabetes mellitus, PG = primary glomerulonephritis, SG = secondary glomerulonephritis, IN = interstitial nephritis, H = hypertension, CHC = cystic/hereditary/congenital, M = miscellaneous.

^B^RV measurements are at recruitment visit for Control and prior to kidney donation for Living Donor patients. 1YR measurements were obtained at 1 year follow-up. The arrows signify the separation of values obtained at RV and 1YR.

^C^Membrane surface area is presented as median (minimum to maximum).

^D^p < 0.05 compared to conventional HD.

^E^p < 0.05 compared to NIPD.

^F^p < 0.05 compared to Home HD SHD.

### Metabolic variation between study groups

**Figure 1:**
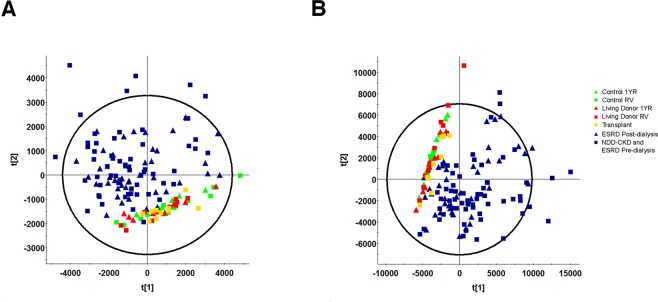
Principal component analysis (PCA) of control (n = 10), living donor (n = 10), kidney transplant (n = 10), non-dialysis dependent CKD (NDD-CKD, n = 20) and ESRD including conventional hemodialysis (conventional HD, n = 10), nocturnal intermittent PD (NIPD, n = 10), frequent nocturnal HHD (n = 5), intermittent nocturnal HHD (n = 5), intermittent conventional HHD (n = 5) and short daily HHD (n = 6) plasma RPLC (**A**) and HILIC (**B**) untargeted metabolomics. Control and living kidney plasma samples were obtained during recruitment visit (RV) and one year follow-up (1YR).

We assessed the overall plasma metabolite variation between study groups by principal component analysis (PCA). Control, living donor and transplant plasma samples clustered together in both reverse phase liquid chromatography (RPLC) and hydrophilic interaction liquid chromatography (HILIC) analysis (Fig. 1A,B, respectively). All other groups did not form a distinct pattern other than separation from control, living donor and transplant groups. A total of 1186 and 1165 features were included in RPLC and HILIC analysis, respectively, after filtering for adducts, isotopes and inconsistent features. To determine the number of metabolites significantly different compared to control, we compared all groups to the control recruitment visit (RV) group by Kruskal-Wallis ANOVA. Significant differences were found in 735 RPLC and 762 HILIC features respectively (p < 0.05, q < 0.05, Supplemental Fig. 1). When plasma from all groups was compared to control RV, dialysis dependent and NDD-CKD patients had a similar number of significantly altered metabolites. NIPD, conventional HD and short daily HHD demonstrated minimal changes in the number of altered metabolites (<20%) between pre and post-dialysis compared to control RV (Supplemental Fig. 1A,B). A greater than 40% decrease was observed in the number of metabolites significantly altered for post-dialysis compared to pre-dialysis samples from both HHD nocturnal groups (frequent nocturnal and intermittent nocturnal, Supplemental Fig. 1C,D). There were no significantly altered metabolites between control RV and control one year follow up (1YR) groups in both HILIC and RPLC methods demonstrating that our analysis was robust in minimizing false positives.

### Plasma biomarkers of reduced kidney function in patients with a single kidney

**Figure 2:**
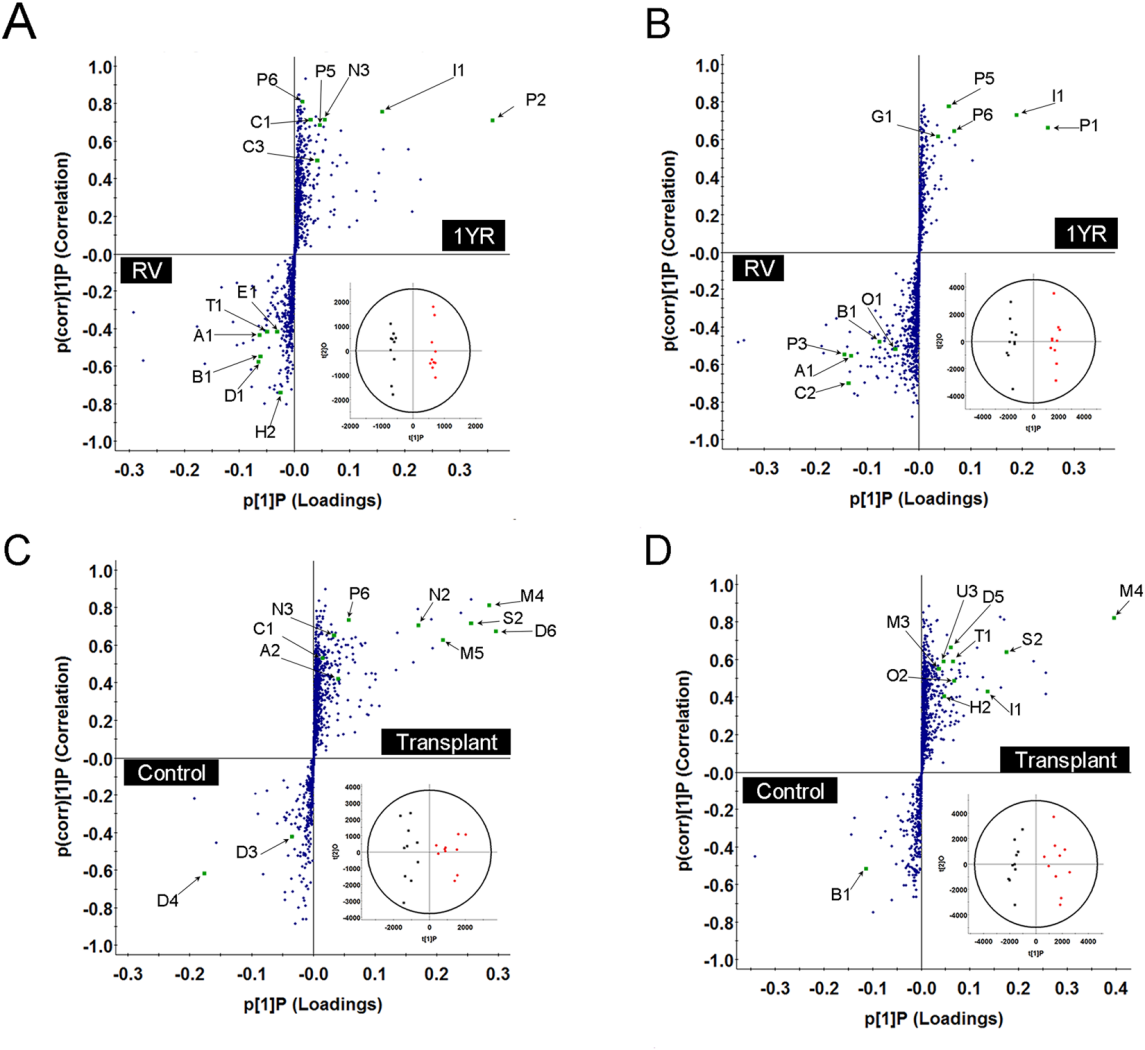
Orthogonal partial least squares discriminant analysis (OPLS-DA) and S-plot projections comparing metabolic features from living kidney donor plasma at recruitment visit (RV, ■) and one-year follow up (1YR, ●) (**A**, RPLC; **B**, HILIC), and control (■) and kidney transplant patient plasma (**C**, RPLC; **D**, HILIC). Feature annotations can be found in Table 2. All labelled features had variable importance in projection (VIP) values > 1 and correlation (pcorr) values > 0.4 or <−0.4, n = 10.

The majority of variation in the PCA containing all groups was influenced by differences between normal renal function and severe renal impairment. Therefore, differences between control and patients with a single kidney (living donor and transplant groups) were further assessed by orthogonal partial least squares discriminant analysis (OPLS-DA). A number of uremic toxins were significantly increased in plasma from living kidney donors after nephrectomy, including N,N,N-trimethyl-L-alanyl-L-proline betaine (TMAP), p-cresyl sulfate, indoxyl sulfate, CMPF, phenylacetylglutamine, pyrocatechol sulfate and creatinine (Fig. 2A, Table 2). Plasma levels of proline betaine, bilirubin, carnitine and several acyl-carnitines were significantly decreased one year post-donation (Fig. 2B). The most robust features defining kidney transplant patient plasma were drug and drug metabolites from antibiotic and immunosuppressant therapy (Fig. 2C). When compared to control subjects, kidney transplant patients also had significantly increased pyrocatechol sulfate, TMAP, uridine and dimethyluric acid levels. Bilirubin was reduced in kidney transplant patient plasma compared to controls.

**Table 2:** Summary of metabolites altered in NDD-CKD and all dialysis modalities when compared to control subjects.

Ion	*t*_R_ (min)	Mass (m/z)	Empirical Formula	Mass Error (ppm)	Identity	ID Level	p-value	q-value	Change after Kidney Donation	Transplant change compared to Control	CKD and Dialysis change compared to Control	CKD and Dialysis change compared to Transplant	Levels Pre vs Post Dialysis					
													Conven-tional HD	NIPD	Frequent Nocturnal HHD	Short Daily HHD	Intermittent Conventional HHD	Intermittent Nocturnal HHD
**A1**	0.94	204.1233	C9H17NO4[H−]	1.5	Acetylcarnitine	2	1.53E-11	7.91E-11	↓				↑	↑	↑	↑	↑	↑
**A2**	1.14	326.0876	C14H16NO8[H−]	0.0	Acetaminophen Glucuronide	1	1.05E-08	4.16E-08		↑	↑	↑	↑		↑	↑	↑	↑
**B1**	2.28	585.2707	C33H36N4O6[H−]	−1.0	Bilirubin	1	9.53E-04	2.12E-03	↓	↓	↓	↓	↓	↓	↓	↓	↓	↓
**B2**	2.67	481.2434	C25H37O9[H−]	−0.7	Hydroxyandro-sterone-glucuronide	3	3.32E-14	2.12E-13			↑		↑		↑	↑	↑	↑
**B3**	1.28	232.1545	C11H22NO4[H+]	−1.7	Butyryl-L-Carnitine	2	3.18E-13	2.11E-12					↑		↑	↑	↑	↑
**C1**	0.55	114.0665	C4H6N3O[H−]	−1.8	Creatinine	1	1.03E-18	8.76E-17	↑		↑	↑	↑		↑	↑	↑	↑
**C2**	0.53	162.1126	C7H16NO3[H+]	−2.5	L-Carnitine	1	2.07E-17	5.85E-16			↓							
**C3**	2.47	239.0917	C12H15O5[H−]	−1.3	CMPF	1	1.28E-02	2.27E-02	↑									
**D1**	2.22	314.2325	C17H32NO4[H+]	−1.9	Decenoylcarnitine	2	1.34E-05	3.61E-05	↓				↑		↑	↑	↑	↑
**D2**	2.30	230.9964	C8H7O6S[H−]	0.0	Dihydroxyaceto-phenone Sulfate	3	7.04E-15	5.43E-14		↑	↑		↑	↑	↑	↑	↑	↑
**D3**	6.44	327.2321	C22H31O2[H−]	−0.9	Docosahexaenoic Acid (DHA)	1	1.21E-07	4.10E-07		↓	↓							
**D4**	2.55	367.1577	C19H27O5S[H−]	−0.5	Dehydroisoandro-sterone sulfate	3	3.28E-05	8.56E-05										
**D5**	2.71	195.0516	C7H7NO4O3[H+]	−1.0	Dimethyluric acid	1	3.42E-07	8.62E-07		↑			↑	↑	↑	↑	↑	↑
**E1**	3.82	426.3576	C25H47NO4[H−]	0.0	Elaidic carnitine	2	8.60E-03	1.58E-02	↓									
**G1**	2.08	226.0172	C5H9NO7P[H−]	24.3	Glutamyl phosphate	2	2.03E-06	4.85E-06			↑							
**H1**	1.60	178.0503	C9H8NO3[H−]	0.6	Hippuric Acid	1	2.61E-14	1.73E-13			↑	↑	↑		↑	↑	↑	↑
**H2**	1.04	137.0460	C5H3N4O[H−]	0	Hypoxanthine	1	1.80E-11	9.24E-11	↓	↑		↑						
**H3**	1.18	227.9964	C8H6NO5S[H−]	−1.3	5-Hydroxy-6-indolyl-O-sulfate	2	1.29E-13	9.34E-13			↑	↑						
**H4**	1.22	246.007	C8H8NO6S[H−]	−1.2	Hydroxy acetami-nophen sulfate	2	2.26E-09	9.62E-09					↑		↑	↑	↑	↑
**I1**	1.71	212.0018	C8H6NO4S[H−]	0	Indoxyl Sulfate	1	4.18E-16	6.28E-15	↑		↑	↑						
**M1**	1.09	181.0359	C6H5N4O3[H−]	−1.7	1-Methyluric Acid	1	1.21E-13	8.82E-13			↑	↑	↑		↑	↑	↑	↑
**M2**	2.91	290.1600	C13H23NO6[H+]	−1.0	3-Methylglutary-lcarnitine	2	1.37E-16	2.32E-15			↑	↑						
**M3**	4.98	170.0928	C7H12N3O2[H+]	−0.6	1-Methylhistidine	1	2.87E-15	2.55E-14		↑	↑	↑						
**M4**	1.95	495.1500	C23H27O12[H−]	−0.6	Mycophenolic Acid Glucuronide	2	2.17E-06	6.42E-06		↑								
**M5**	2.64	319.1181	C17H19O6[H−]	−0.3	Mycophenolic acid	2	1.68E-14	1.17E-13		↑								
**N1**	1.09	153.0660	C7H9N2O2[H+]	0.0	2-PY	1	2.06E-16	3.59E-15			↑	↑	↑		↑	↑	↑	↑
**N2**	2.03	294.0548	C12H12N3O4S[H−]	−0.3	N4-Acetylsulfa-methoxazole	1	4.15E-09	1.71E-08		↑								
**N3**	1.05	229.1549	C11H21N2O3[H+]	−1.3	N,N,N-trimethyl-L-alanyl-L-proline (TMAP)	1	6.06E-19	6.53E-17	↑	↑	↑		↑	↑	↑	↑	↑	↑
**O1**	1.98	310.2012	C15H29NO4[Na+]	5.5	Octanoyl Carnitine	1	1.02E-13	7.51E-13			↑	↑	↑	↑	↑	↑	↑	↑
**O2**	1.06	260.0228	C9H10NO6S[H−]	−0.4	O-Sulfo-L-Tyrosine	1	1.67E-18	9.93E-17			↑	↑	↑		↑	↑	↑	↑
**O3**	0.67	203.0014	C7H7O5S[H−]	1.5	O-methoxy-catechol-O-sulfate	3	3.57E-09	1.07E-08		↑	↑		↑		↑	↑	↑	↑
**P1**	1.67	283.0822	C13H15O7[H−]	1.4	P-Cresyl Glucuronide	1	8.58E-12	4.60E-11			↑		↑		↑	↑	↑	↑
**P2**	1.79	187.0066	C7H7O4S[H−]	0.5	P-Cresyl Sulfate	1	2.22E-08	8.38E-08	↑		↑	↑						
**P3**	3.71	144.1025	C7H14NO2[H+]	0.7	Proline Betaine	1	2.00E-06	4.79E-06			↑	↑	↑		↑	↑	↑	↑
**P4**	1.63	172.9908	C6H5O4S[H−]	0.6	Phenyl Sulfate	1	4.97E-13	3.15E-12			↑		↑	↑	↑	↑	↑	↑
**P5**	1.57	263.1033	C13H15N2O4[H−]	0.4	Phenylacety-lglutamine	1	7.18E-18	2.66E-16	↑	↑	↑	↑	↑	↑	↑	↑	↑	↑
**P6**	1.59	188.9856	C6H5O5S[H−]	−1.1	Pyrocatechol Sulfate	1	1.55E-13	1.11E-12	↑	↑	↑	↑	↑		↑	↑	↑	↑
**S1**	1.31	216.9806	C7H5O6S[H−]	−0.5	5-Sulfosalicylic Acid	2	1.40E-14	1.21E-13			↑	↑	↑	↑	↑	↑	↑	↑
**S2**	2.02	254.0598	C10H12N3O3S[H+]	−0.4	Sulfamethoxazole	1	1.43E-04	3.50E-04		↑								
**T1**	2.65	498.2884	C26H44NO6S[H−]	−2.2	Taurodeoxy (cheno)cholic Acid	2	1.96E-03	4.11E-03	↓	↑								
**T2**	2.15	514.2833	C26H44NO7S[H−]	1.6	Taurocholic Acid	1	3.44E-03	7.02E-03										
**T3**	1.49	291.1454	C14H19N4O3[H+]	−1.0	Trimethoprim	1	3.82E-10	1.75E-09		↑								
**U1**	2.88	379.0334	C10H12N4O10P[H−]	11.3	Urate D-Ribonu-cleotide	2	1.68E-14	1.17E-13				↑	↑		↑	↑	↑	↑
**U2**	3.86	167.0203	C5H3N4O3[H−]	−1.2	Uric Acid	1	4.43E-10	1.46E-09					↑	↑	↑	↑	↑	↑
**U3**	3.56	243.0616	C9H12HNO6[H−]	−0.4	Uridine	1	4.88E-16	6.05E-15		↑	↑	↑	↑		↑	↑	↑	↑

**Figure 3:**
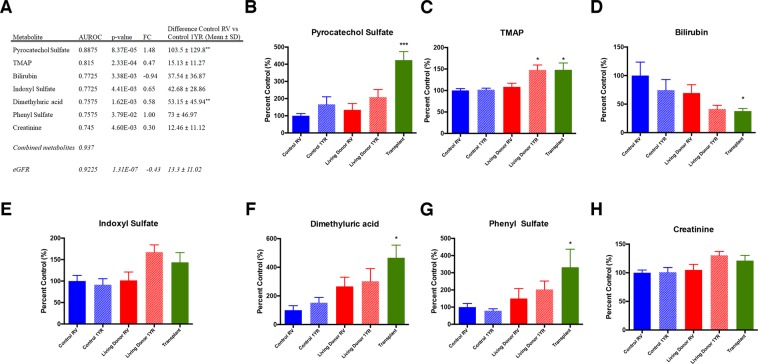
Diagnostic performance of plasma metabolites found to more accurately predict decreased kidney function in patients with a single kidney than creatinine. Metabolites are ranked by area under the receiver operating characteristic (AUROC) curve and p-value (**A**). The mean differences between control RV and control 1YR were compared to eGFR. Plasma levels of pyrocatechol sulfate (%CV = 9.0) (**B**), N,N,N-trimethyl-L-alanyl-L-proline betaine (TMAP, %CV = 6.2, **C**), bilirubin (%CV = 8.5, **D**), phenyl sulfate (%CV = 6.5, (**E**) and dimethyluric acid (%CV = 8.4, (**F**) are presented for control RV, control 1YR, living donor RV, living donor 1YR and transplant patients. Data is presented as mean ± SEM, *p < 0.05, **p < 0.01, FC = fold change.

**Figure 4:**
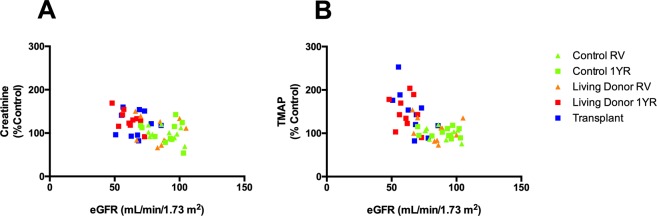
Association of creatinine (**A**) and TMAP (**B**) with eGFR plasma samples from control plasma at recruitment visit (RV), one-year follow up (1YR), living donor RV, living donor 1YR and kidney transplant patients, n = 10 for all groups. eGFR was calculated using the MDRD method.

To assess the performance of metabolites significantly altered in living donor and transplant patients as biomarkers of altered kidney function, we performed receiver operating characteristic analysis. TMAP, pyrocatechol sulfate, indoxyl sulfate, bilirubin, phenyl sulfate, and dimethyluric acid more accurately predicted decreased kidney function in patients with a single kidney than creatinine (Fig. 3). TMAP and creatinine demonstrated the lowest variation between control RV and control 1YR. Moreover, only TMAP was significantly increased in both living kidney donor and kidney transplant patient plasma when compared to control RV (*P* < 0.05, Fig. 3B). TMAP is also more sensitive than creatinine as demonstrated when comparing both metabolites with eGFR (Fig. 4). When assessing the performance of all metabolites in Fig. 3, the combined AUROC was 0.937 (0.816–1.00), which was a slight improvement over eGFR.

### Plasma biomarkers of end-stage renal disease

To assess plasma metabolic disturbances in various dialysis modalities as well as NDD-CKD, we compared NDD-CKD as well as pre-dialysis conventional HD, NIPD and frequent nocturnal HHD to subjects with normal kidney function. Comparisons for each CKD group to control patients were well modelled by OPLS-DA (Supplemental Table 2). A total of 24 metabolites were found to be significantly increased in all kidney disease groups compared to normal kidney function. Many of these metabolites were gut-derived and indoxyl sulfate and p-cresyl sulfate had the largest increase in plasma levels for all groups when compared to controls (Supplemental Fig. 2). The majority of these metabolites were also sulfate containing compounds including O-sulfoyl-tyrosine, 5-hydroxy-6-indolyl-O-sulfate, phenyl sulfate, and pyrocatechol sulfate. 5-Hydroxy-6-indolyl-O-sulfate was newly identified as a potential uremic toxin (Table 2). Circulating carnitine, bilirubin, dehydroisoandrosterone sulfate and docosahexaenoic acid (DHA) levels were decreased in CKD plasma (Table 2).

**Figure 5:**
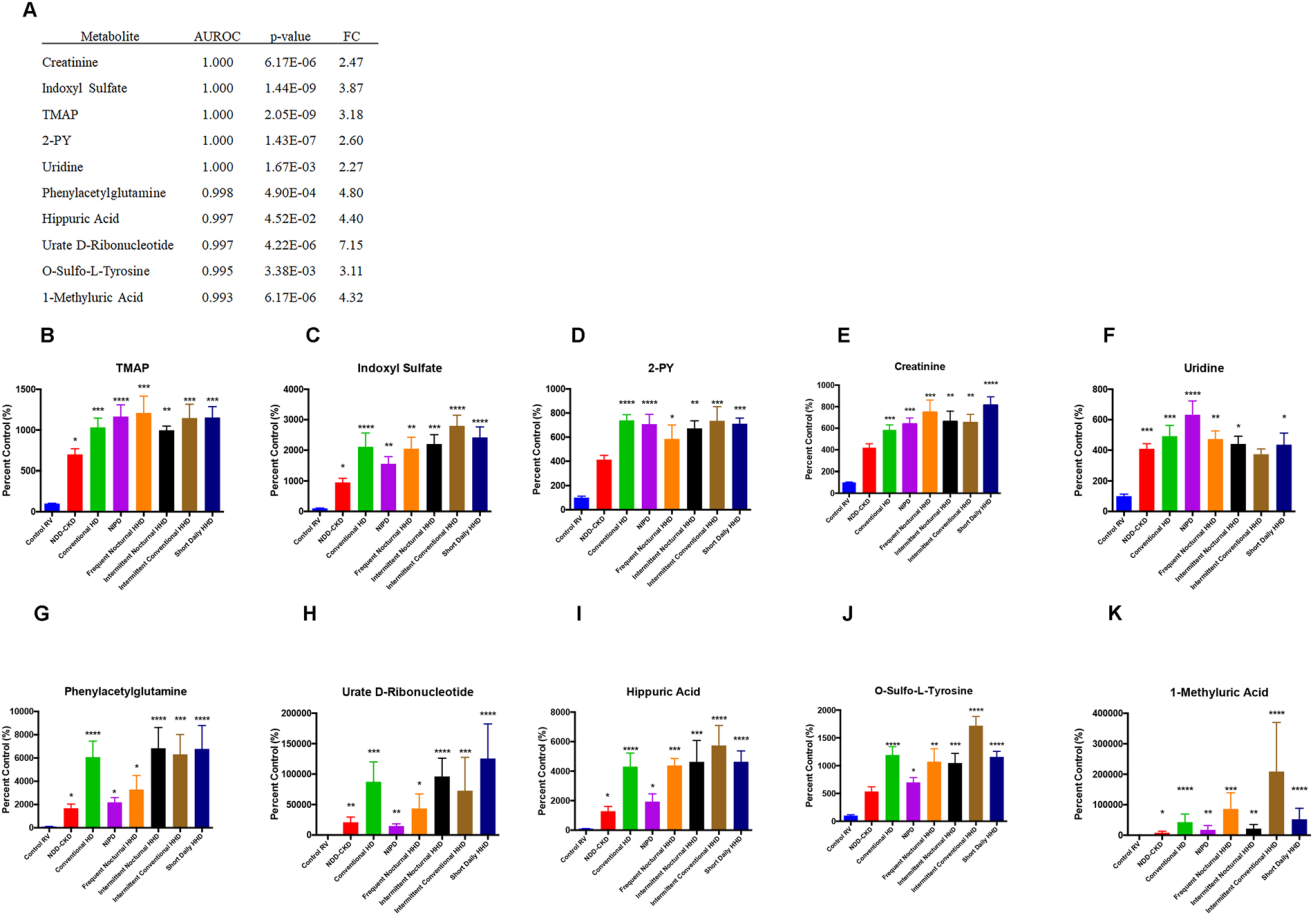
Diagnostic performance of the top 10 plasma metabolites found to accurately predict decreased kidney function in ESRD. Metabolites are ranked by area under the receiver operating characteristic (AUROC) curve and p-value (**A**). Plasma levels of indoxyl sulfate (%CV = 5.9, **B**), N,N,N-trimethyl-L-alanyl-L-proline betaine (TMAP, %CV = 6.2, **C**), N-methyl-2-pyridone-5-carboxamide (2-PY, %CV = 7.1, **D**), creatinine (%CV = 9.0, **E**), uridine (%CV = 8.3, **F**), phenylacetylglutamine (%CV = 5.4, **G**), urate d-ribonucleotide (%CV = 7.2, **H**), hippuric acid (%CV = 7.4, **I**), o-sulfo-L-tyrosine (%CV = 8.1, **J**) and 1-methyluric acid (%CV = 7.2, **K**) are presented for control RV (n = 10), non-dialysis dependent CKD (NDD-CKD, n = 20), conventional hemodialysis (conventional HD, n = 10), nocturnal intermittent PD (NIPD, n = 10), frequent nocturnal HHD (n = 5), intermittent nocturnal HHD (n = 5), intermittent conventional HHD (n = 5), and short daily HHD (n = 6). Data is presented as mean ± SEM, n = 10, *p < 0.05, **p < 0.01, FC = fold change.

Univariate ROC analysis was performed on each metabolite found to be significantly increased in NDD-CKD and pre-ESRD patient plasma compared to normal kidney function. TMAP, creatinine, indoxyl sulfate, N-methyl-2-pyridone-5-carboxamide (2-PY) and uridine were 100% accurate in predicting ESRD (Fig. 5). The ten most highly predictive biomarkers of ESRD also included phenlyacetylglutamine, hippuric acid, urate D-ribonucleotide, O-sulfotyrosine and 1-methyluric acid. TMAP appeared to be a more sensitive biomarker than creatinine and was 7.0-fold and 10.3-fold increased in NDD-CKD and conventional HD, respectively, compared to controls. Creatinine was only increased 4.2-fold and 5.8-fold in NDD-CKD and conventional HD compared to controls in the same patients. Correlation was also performed for key significantly altered metabolites with subject age. Dehydroisoandrosterone sulfate and bilirubin were negatively correlated with age (q < 0.05). 2-PY, uridine, p-cresyl sulfate, proline betaine, octanoyl carnitine and 1-methylhistidine were positively correlated with age. Metabolite differences between sexes were also determined and no significant differences were found.

### Plasma biomarkers of hemodialytic clearance

To assess the dialytic clearance of plasma metabolites we performed untargeted metabolomics analysis on pre and post-dialysis plasma samples from patients on conventional HD, NIPD, short daily HHD, frequent nocturnal HHD, intermittent conventional HHD and intermittent nocturnal HHD. Hemodialysis modalities resulted in the net clearance of many similar metabolites and several more than NIPD (Supplemental Figs 4 and 5). Decreased plasma levels of several gut-derived uremic toxins were also found in post-dialysis hemodialysis samples. In NIPD patients, only the gut-derived uremic toxins phenylacetylglutamine and phenyl sulfate were significantly decreased in plasma after dialysis. All hemodialysis modalities caused a significant decrease in the same 25 plasma metabolites (Table 2); however, only 9 of these metabolites were also cleared by NIPD. Therefore, we focused our analysis on hemodialytic clearance.

**Figure 6:**
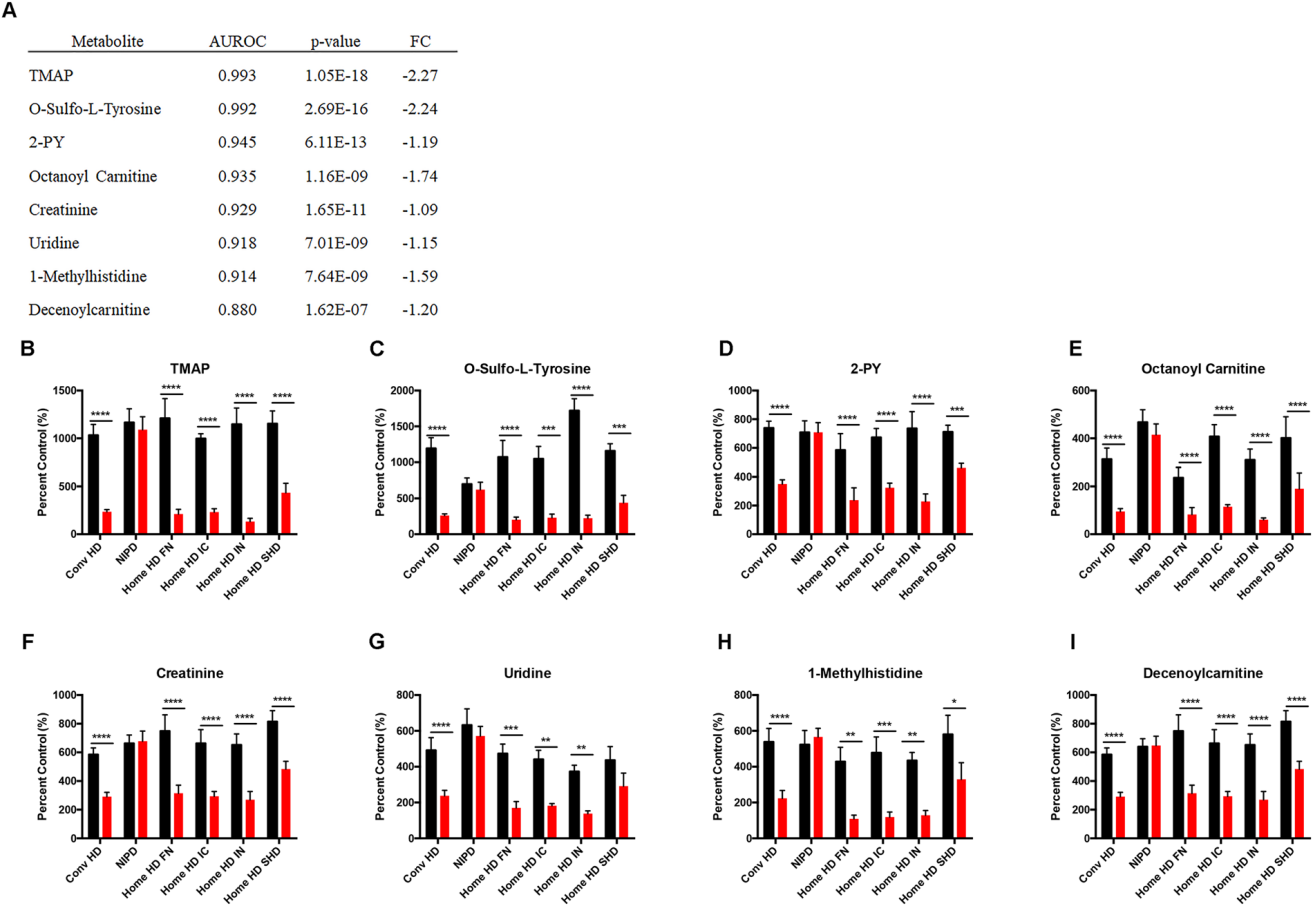
Plasma metabolites most efficiently cleared by hemodialysis. Metabolites are ranked by area under the receiver operating characteristic (AUROC) curve and p-value (**A**). Pre and post-dialysis plasma levels of N,N,N-trimethyl-L-alanyl-L-proline betaine (TMAP, %CV = 6.2, **B**), o-sulfo-L-tyrosine (%CV = 8.1, **C**), N-methyl-2-pyridone-5-carboxamide (2-PY, %CV = 7.1, **D**), octanoyl carnitine (%CV = 12.0, **E**), creatinine (%CV = 9.0, **F**), uridine (%CV = 8.3, **G**), 1-methylhistidine (%CV = 12.9, **H**) and decenoyl carnitine (%CV = 6.1, **I**) are presented for control RV (n = 10), non-dialysis dependent CKD (NDD-CKD, n = 20), conventional hemodialysis (conventional HD, n = 10), nocturnal intermittent PD (NIPD, n = 10), frequent nocturnal HHD (n = 5), intermittent nocturnal HHD (n = 5), intermittent conventional HHD (n = 5), and short daily HHD (n = 6). Data is presented as mean ± SEM, and analyzed by repeated measures ANOVA with Sidak’s correction *p < 0.05, **p < 0.01, FC = fold change.

To assess potentially novel biomarkers of dialytic clearance we combined all hemodialysis modalities and performed ROC analysis. TMAP was the most consistently cleared metabolite resulting in a 62–88% decrease in post-dialysis plasma levels in hemodialysis modalities (AUC: 0.993, Fig. 6A). Conversely, creatinine plasma levels only decreased 41–58% after hemodialysis (AUC: 0.929, Fig. 6B). Other metabolites significantly cleared by hemodialysis included o-sulfotyrosine, 2-PY, octanoyl carnitine, uridine, 1-methylhistidine and decenoylcarnitine (Fig. 6).

**Figure 7:**
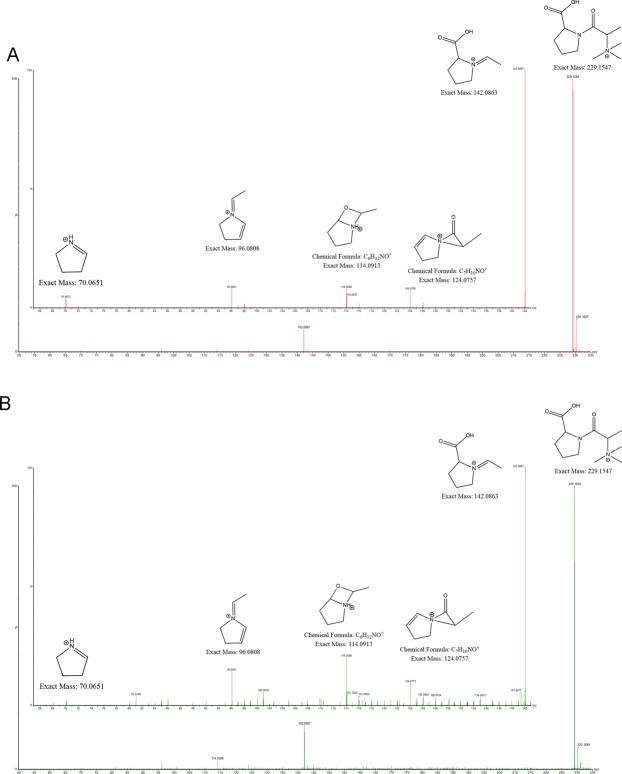
Synthesized N,N,N-trimethyl-L-alanyl-L-proline betaine (TMAP, (**A**) and patient plasma sample (**B**) spectrum in MS^E^ (collision energy ramp of 15–50 V, **A**). TMAP parent ion 229 m/z and fragments 142 m/z, 96 m/z and 70 m/z demonstrated in MS^E^ identify the structure of TMAP (30).

Finally, the identity of TMAP was confirmed by comparing feature fragmentation pattern and retention time in plasma samples with synthesized TMAP (Fig. 7).

## Discussion

This study reports untargeted metabolomics to identify metabolic plasma biomarkers of reduced kidney function in early CKD, ESRD and hemodialytic clearance. In our initial analysis, plasma samples from patients with a single kidney were similar to control samples but distinctly different than NDD-CKD and all dialysis groups (Fig. 1). These striking differences demonstrate that substantial kidney function can be maintained with a single kidney and clearly show the benefits of kidney transplantation on the plasma metabolic profile when compared to dialysis.

The current guidelines for diagnosis and staging of CKD rely on creatinine as a biomarker to estimate GFR^20^. However, creatinine has limited sensitivity and may not be altered until kidney function has decreased by 50% (eGFR < 60 ml/min per 1.73 m^2^)^21^. Although creatinine was a consistent biomarker of reduced kidney function in ESRD, several metabolites including TMAP and pyrocatechol sulfate outperformed creatinine as biomarkers of reduced kidney function. Despite the limitations of creatinine as an early stage CKD biomarker, eGFR was more accurate than all other individual plasma biomarkers measured in the study (Fig. 3A). However, the range of control and single kidney patient eGFR appeared to be less dependent on serum creatinine than other eGFR parameters (Fig. 4A). Indeed, the current equations for estimating GFR including MDRD and CKD-EPI are inaccurate in earlier stages of CKD^22^. Therefore, applying common eGFR parameters such as age, gender and race along with more predictive plasma biomarkers identified in this study (e.g. TMAP, pyrocatechol sulfate) may allow for the development of improved equations to estimate GFR in earlier stages of CKD. Moreover, a combination of biomarkers identified in this study may provide an improved metabolic fingerprint of early stage CKD.

The structure of TMAP was recently identified but its use as a biomarker of kidney function has not been reported^23^. Moreover, the variation in TMAP over one year for Control RV and Control 1YR was not significantly different than eGFR (Fig. 3A). Samples from the same patients were drawn one year apart, which suggests that TMAP may be a consistent and robust marker of kidney function.

The gut-derived metabolites, indoxyl sulfate and p-cresyl sulfate, were the most abundant in NDD-CKD and all dialysis modalities when compared to control subjects (Supplemental Fig. 2). Furthermore, indoxyl sulfate and p-cresyl sulfate were the most significantly increased metabolites one year after kidney donation in living donors (Fig. 2A). Indoxyl sulfate and p-cresyl sulfate have been correlated with cardiovascular events^9^. Aortic calcification and left ventricle systolic dysfunction are also associated with high levels of indoxyl sulfate^12,24^. In addition, high levels of unbound plasma p-cresyl sulfate have been shown to increase the risk of all-cause mortality^11^. Therefore, indoxyl sulfate and p-cresyl sulfate are strong predictors of cardiovascular mortality in ESRD and may increase the risk of cardiovascular disease in living kidney donors. Since levels are increased after only one year following donation, studies assessing long-term complications of kidney donation should assess the change of indoxyl sulfate and p-cresyl sulfate concentration.

As expected, dialytic clearance of several metabolites was demonstrated when comparing pre- and post-hemodialysis plasma samples. Patients receiving nocturnal hemodialysis had the greatest difference between pre- and post-dialysis samples (Fig. 1E,F); however, pre-dialysis levels for several metabolites were similar across hemodialysis groups regardless of protein binding. These data are consistent with a recent study evaluating the effect of dialysis frequency on steady-state uremic toxin levels, which suggests that steady-state metabolite levels may also be dependent on other factors including metabolite production^25^. Interestingly, fewer metabolites underwent dialytic clearance during NIPD than all hemodialysis modalities. Unlike hemodialysis, NIPD relies more on passive diffusion of solutes, likely decreasing metabolite clearance. However, the dialysis dose, defined by Kt/V, was similar for NIPD compared to hemodialysis modalities. Therefore, the use of several metabolites may provide a more informative assessment of dialysis and aid clinicians when choosing an appropriate modality for their patients.

TMAP was the most consistently cleared metabolite by all hemodialysis modalities in our untargeted metabolomics analysis. Although the biological origin of TMAP has not been identified, we suggest that TMAP may be produced from degradation of myosin light chain (MYL) proteins. N,N,N-trimethylalanine is mainly found in myosin light chain (MYL) proteins and in each of the four MYL isoforms (MYL1, MYL2, MYL3, and MYL4), the c-terminus of N,N,N-trimethylalanine forms a peptide bond with proline^26^. Therefore, MYL protein degradation may be responsible for the release of TMAP. Further study is necessary to determine the biological origin and potential physiological effects of TMAP.

There are several limitations to this study. The sample size for each group was 5 to 20 patients, which limited our ability to control age and sex across groups. The home hemodialysis patient groups were most limited due to the small number of patients prescribed each home hemodialysis modality. In addition, relative quantification was performed for all features in the study followed by confirmation with known standards.

In conclusion, this study provides evidence for novel plasma metabolic biomarkers of reduced kidney function in patients with a single kidney and ESRD as well as insight into the dialytic clearance of plasma metabolites in various hemodialysis modalities. Our key finding is the identification of TMAP as a potential novel plasma biomarker of reduced kidney function in early CKD, ESRD and hemodialytic clearance. Future study of TMAP in a larger patient population will be necessary to evaluate its use as a biomarker in the metabolic fingerprint of kidney disease. The potential impact of comorbidities, medications and other factors on TMAP interindividual variability also remain to be determined. Furthermore, a larger clinical study can determine the performance of TMAP in assessing CKD progression, inflammation and tubular injury including c-reactive protein, kidney injury molecule-1, IL-18, monocyte chemotactic protein-1 and YKL-40^27^ injury. Our results suggest that TMAP, possibly along with other metabolites, may allow for the derivation of a new equation to provide more accurate estimates of GFR. Furthermore, we demonstrate the pronounced metabolic differences between transplantation and dialysis therapy and the notable inferior NIPD dialytic metabolite clearance compared to hemodialysis.

## Methods

### Study participants

Conventional HD, NIPD, short daily HHD, frequent nocturnal HHD, intermittent conventional HHD, intermittent nocturnal HHD, kidney transplant, NDD-CKD, living kidney donors and controls were recruited from the Southwestern Ontario Regional Renal Program between 2010 and 2015. Eligible participants were over the age of 18 and excluded if they had evidence of gastrointestinal disease (not including gastroesophageal reflux disease). Patients maintained their regular diet and were not asked to fast prior to the study. Participant demographic information was recorded on the day of sample collection from electronic health records. eGFR was calculated using the modified diet in renal disease (MDRD) equation. This study was approved by the Western University Health Sciences Research Ethics Board and was conducted according to the Declaration of Helsinki principles. Written informed consent was received from patients prior to inclusion in the study.

### Sample collection

For dialysis participants, blood was collected in EDTA coated tubes immediately prior to and following the dialysis session. CKD and transplant blood samples were collected during routine clinic visits. Blood from living kidney donors and matched control subjects were collected during a recruitment visit and one year following kidney donation or recruitment visit, respectively. Blood samples were centrifuged at 2000 × G to obtain plasma. Plasma was aliquoted and stored at −80C.

### Sample processing

For metabolomics analysis, plasma samples were thawed at 4 °C and proteins were precipitated using a 3:1 ratio of ice-cold acetonitrile to sample as previously described^28,29^. The acetonitrile contained α-aminopimelic acid (100 μM, Toronto Research Chemicals) and chlorpropamide (2.5 μM, Sigma) as internal standards for HILIC and RPLC, respectively. Metabolites were separated by HILIC and RPLC followed by Time-of-Flight mass spectrometry on a Waters Xevo-G2S QTof/MS.

### Chromatography and mass spectrometry for metabolomic profiling

For HILIC analysis, 1 μL of sample was injected onto a Waters ACQUITY UPLC BEH Amide (1.7 μm particle size, 100 × 2.1 mm). Samples underwent a subsequent 1:5 dilution in water for RPLC analysis and 5 μL was injected onto a Waters ACQUITY UPLC HSS T3 column (1.8 μm particle size, 100 × 2.1 mm). Both columns were maintained at 45 °C and mobile phase flow was set to 0.45 mL/min using a Waters ACQUITY UPLC I-Class system (Waters, Milford, MA). The mobile phase consisted of water containing 0.1% formic acid (A) and acetonitrile containing 0.1% formic acid (B). Mobile phase conditions for each column are described in Table S1. Time-of-Flight mass spectrometry was carried out using a Waters Xevo-G2S QTof/MS as previously described^28,29^. Briefly, capillary and cone voltage were set at 2 kV and 40 V, respectively. The source temperature was 150 °C and the desolvation temperature was maintained at 600 °C. Nitrogen gas for desolvation and the cone were set at 1200 L/h and 50 L/h, respectively. An MS^E^ method was used to acquire ions in the range of 50–1200 m/z alternating between MS1 (no collision energy) and MS2 (collision energy ramp of 15–50 V) with a scan time of 0.1 s. Leucine-enkephalin (100 ng/L) was used as a lockmass set to a flow rate of 10 μl/min. The lockmass was acquired every 10 seconds and averaged over 3 scans to ensure mass accuracy throughout the run.

### Quality control and batch organization

All samples were run as a single batch for each chromatographic condition and ionization mode (e.g. RPLC-negative ESI, HILIC-positive ESI, etc.). A pooled sample was generated by combining the same volume of all samples into a single vial. The injection of samples was randomized and the pooled sample was injected every 6 samples.

### Data processing

Masslynx raw data files were converted to mzData files as previously described^29^ in R version 3.2.0. Isotopologue Parameter Optimization (IPO version 1.7.4) was performed on pooled injections to optimize peak-picking, retention time correction and grouping parameters prior to XCMS (version 1.42) analysis of samples^30,31^. The CAMERA package (version 1.26.0) was used to annotate adducts, isotopes and metabolites that ionize in both positive and negative mode^32^. Resulting features were normalized to internal standard. Features in pooled samples that exhibited a relative standard deviation greater than 30% over each sample batch were considered unreliable and removed from further analysis. Feature groups of adducts and isotopes were created and the feature with the maximum intensity in each group was chosen for analysis. For features that ionized in both positive and negative ESI modes, the more sensitive ion was used. Subsequently, positive and negative ESI mode data sets were combined for statistical analysis.

### Statistics

#### Univariate statistics

Data was analyzed using the Kruskal-Wallis ANOVA followed by Dunn’s post-hoc test with the R statistics and DescTools packages. P values were adjusted according to the Benjamini Hochberg procedure and q < 0.05 was considered significantly different. For pre and post-dialysis comparisons, a repeated measures ANOVA with Sidak’s correction was performed.

#### Multivariate statistics

The data was processed by mean centering and pareto scaling in EZinfo 2.0 (Umetrics, Umeå, Sweden). PCA was used to visualize general trends in the data. Metabolic differences between individual groups were assessed by orthogonal partial least squares discriminant analysis (OPLS-DA). A multilevel PLS-DA was used for paired analysis to report the within patient differences for pre- and post-dialysis and pre and post kidney donation as previously described^33^. Goodness-of-fit and predictive ability was determined based on R2Y and Q2Y values, respectively. Features with variable importance in projection (VIP) values > 1 and correlation (pcorr) values > 0.4 were considered significant and chosen as putative markers for identification.

#### Associations with age and sex

Potential correlations with metabolite level and age were determined using Pearson correlation coefficients and corrected by Benjamini Hochberg procedure. Metabolite level association with sex was determined.

### Metabolite identification

Features considered for identification were searched in METLIN, Human Metabolome Database (HMDB), Lipid Maps and Chemspider. Spectral matching of feature fragmentation patterns to putative compound fragmentation was determined using MassFragment®. Standards were purchased to confirm metabolite identities, if available. Metabolite identification levels are presented according to the Chemical Analysis Working Group, as previously described^34^.

#### Receiver operating characteristic analysis

Metabolites found to be significantly different by multivariate statistics were assessed by univariate ROC analysis using MetaboAnalyst^35^. For biomarker ROC analysis, control RV and living donor RV samples were combined and classified as normal kidney function. The performance of each significantly altered metabolite as a biomarker of early stage CKD was compared to the performance of eGFR. ROC analysis was also performed on metabolites significantly increased in ESRD and significantly cleared by hemodialytic clearance.

## Supplementary information

Supplementary Info

## Data Availability

Metabolite data tables will be made freely available upon request.
