# Supplementary material for: Untargeted metabolomics reveals N, N, N-trimethyl-L-alanyl-L-proline betaine (TMAP) as a novel biomarker of kidney function
Source: Sci Rep. 2019 May 2;9:6831. doi: 10.1038/s41598-019-42992-3 (PMC6497643; doi:10.1038/s41598-019-42992-3)
Supplement: Supplementary file 1 — Supplementary Info [file 41598_2019_42992_MOESM1_ESM.pdf]

SUPPLEMENTARY INFORMATION

Untargeted metabolomics reveals N,N,N-trimethyl-L-alanyl-L-proline betaine (TMAP) as a novel biomarker of kidney function

Thomas J. Velenosi PhD, Benjamin K.A. Thomson MD, Nicholas C. Tonial BMSc, Adrien A.E. RaoPeters, Megan A. Mio BMSc, Gilles A. Lajoie, Amit X. Garg MD PhD, Andrew A. House MD, and Bradley L. Urquhart PhD

Supplemental Table 1. Chromatographic conditions for RPLC and HILIC separation of metabolites.

	Time (min)	0.1% Formic Acid in Water (%)	0.1% Formic Acid in Acetonitrile (%)	Curve
**RPLC**	0.00	99	1	6
	2.00	40	60	6
	6.00	15	85	6
	8.00	1	99	6
	10.00	99	1	6
	11.00	99	1	6
**HILIC**	0.00	1	99	6
	0.50	1	99	6
	6.00	50	50	6
	8.00	70	30	6
	8.51	1	99	6

Supplemental Table 2. Goodness of fit (R2Y) and predictive ability (Q2Y) for OPLS-DA and multilevel PLS-DA analysis in this study.

Figure	Comparison	R2Y	Q2Y
**Fig. 2A**	Living Donor RV vs 1YR Plasma RPLC	0.993	0.664
**Fig. 2B**	Living Donor RV vs 1YR Plasma HILIC	0.992	0.314
**Fig. 2C**	Control vs Transplant RPLC	0.616	0.883
**Fig. 2D**	Control vs Transplant HILIC	0.933	0.592
**Suppl. Fig. 1A**	Control vs CKD RPLC	0.876	0.653
**Suppl. Fig. 1B**	Control vs CKD HILIC	0.799	0.634
**Suppl. Fig. 1C**	Control vs Conv HD Pre RPLC	0.938	0.623
**Suppl. Fig. 1D**	Control vs Conv HD Pre HILIC	0.875	0.520
**Suppl. Fig. 1E**	Control vs NIPD Pre RPLC	0.946	0.730
**Suppl. Fig. 1F**	Control vs NIPD Pre HILIC	0.863	0.629
**Suppl. Fig. 1G**	Control vs Frequent Nocturnal HHD Pre RPLC	0.974	0.720
**Suppl. Fig. 1H**	Control vs Frequent Nocturnal HHD Pre HILIC	0.963	0.864
**Suppl. Fig. 2A**	Transplant vs CKD RPLC	0.870	0.616
**Suppl. Fig. 2B**	Transplant vs CKD HILIC	0.738	0.513
**Suppl. Fig. 2C**	Transplant vs Conventional HD Pre RPLC	0.947	0.669
**Suppl. Fig. 2D**	Transplant vs Conventional HD Pre HILIC	0.819	0.488
**Suppl. Fig. 2E**	Transplant vs NIPD Pre RPLC	0.948	0.765
**Suppl. Fig. 2F**	Transplant vs NIPD Pre HILIC	0.819	0.596
**Suppl. Fig. 2G**	Transplant vs Frequent Nocturnal HHD Pre RPLC	0.983	0.731
**Suppl. Fig. 2H**	Transplant vs Frequent Nocturnal HHD Pre HILIC	0.945	0.837
**Suppl. Fig. 3A**	Conventional HD Pre vs NIPD Pre RPLC	0.974	0.251

**Suppl. Fig. 3B**	Conventional HD Pre vs NIPD Pre HILIC	0.708	0.201
**Suppl. Fig. 3C**	Conventional HD Pre vs Frequent Nocturnal HHD Pre RPLC	0.816	0.252
**Suppl. Fig. 3D**	Conventional HD Pre vs Frequent Nocturnal HHD Pre HILIC	0.796	-0.002
**Suppl. Fig. 3E**	Conventional HD Pre vs NIPD Pre RPLC	0.930	0.594
**Suppl. Fig. 3F**	Conventional HD Pre vs NIPD Pre HILIC	0.875	0.540
**Suppl. Fig. 4A**	Conventional HD Pre vs Post RPLC	0.997	0.770
**Suppl. Fig. 4B**	Conventional HD Pre vs Post HILIC	0.990	0.641
**Suppl. Fig. 4C**	Frequent Nocturnal HHD Pre vs Post RPLC	0.998	0.914
**Suppl. Fig. 4D**	Frequent Nocturnal HHD Pre vs Post HILIC	0.981	0.871
**Suppl. Fig. 4E**	NIPD Pre vs Post RPLC	0.979	0.546
**Suppl. Fig. 4F**	NIPD Pre vs Post HILIC	0.997	0.181
**Suppl. Fig. 5A**	Control vs Intermittent Conventional HHD Pre RPLC	0.973	0.744
**Suppl. Fig. 5B**	Control vs Intermittent Conventional HHD Pre HILIC	0.992	0.908
**Suppl. Fig. 5C**	Control vs Intermittent Nocturnal HHD Pre RPLC	0.984	0.665
**Suppl. Fig. 5D**	Control vs Intermittent Nocturnal HHD Pre HILIC	0.959	0.741
**Suppl. Fig. 5E**	Control vs Short Daily HHD Pre RPLC	0.958	0.759
**Suppl. Fig. 5F**	Control vs Short Daily HHD Pre HILIC	0.963	0.830
**Suppl. Fig. 6A**	Intermittent Conventional HHD Pre vs Post RPLC	0.998	0.853
**Suppl. Fig. 6B**	Intermittent Conventional HHD Pre vs Post HILIC	0.988	0.911
**Suppl. Fig. 6C**	Intermittent Nocturnal HHD Pre vs Post RPLC	0.999	0.959
**Suppl. Fig. 6D**	Intermittent Nocturnal HHD Pre vs Post HILIC	0.996	0.795

**Suppl. Fig. 6E**	Short Daily HHD Pre vs Post RPLC	0.997	0.927
**Suppl. Fig. 6F**	Short Daily HHD Pre vs Post HILIC	0.992	0.719

Supplemental Figure 1

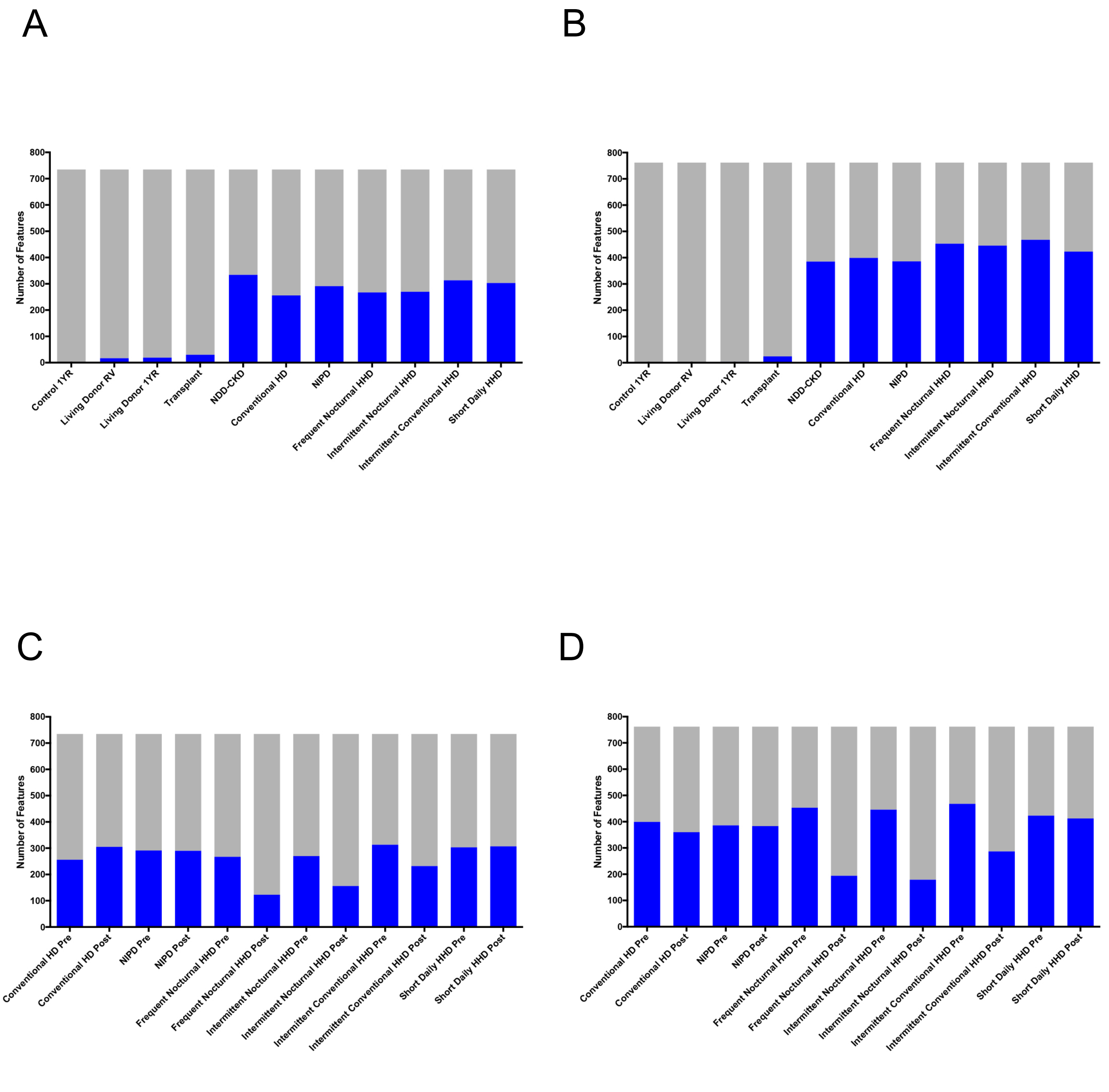

Supplementary Figure 1. Number of features significantly different by Kruskal-Wallis ANOVA ($p<0.05$ and $q<0.05$) followed by Dunn's post-hoc test for RPLC (A) and HILIC (B) analysis (grey bars) and features significantly different than control RV samples (blue bars). Features significantly different than control RV for pre- and post-dialysis samples are also presented from RPLC (C) and HILIC (D) metabolomics analysis. Control and living kidney plasma samples were obtained during recruitment visit (RV) and one year follow-up (1YR).

Supplemental Figure 2

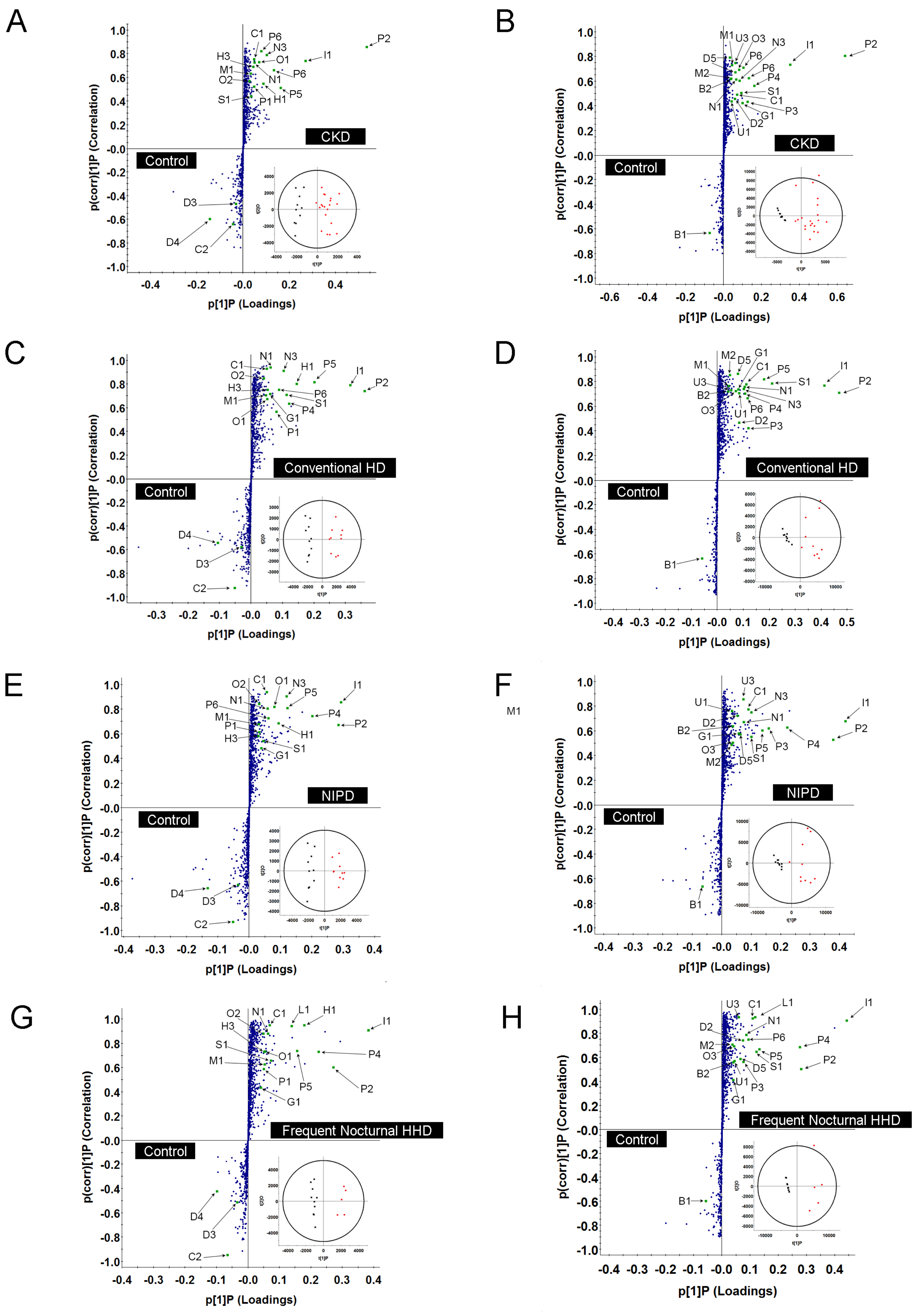

Supplementary Figure 2. Orthogonal partial least squares discriminant analysis (OPLS-DA) and S-plot projections comparing plasma metabolic features from control (■, n = 10) subjects with CKD (●,n = 20, A, RPLC; B, HILIC), conventional hemodialysis (conventional HD, ●, n = 10; C, RPLC; D, HILIC), nocturnal intermittent peritoneal dialysis (NIPD, ●,n = 10; E, RPLC; F, HILIC) and frequent nocturnal HHD (●, n = 5; HD E, RPLC; F, HILIC). Features are annotated (see Table 2 for metabolite list) with variable importance in projection (VIP) values > 1 and correlation (pcorr) values > 0.4 or < -0.4.

Supplemental Figure 3

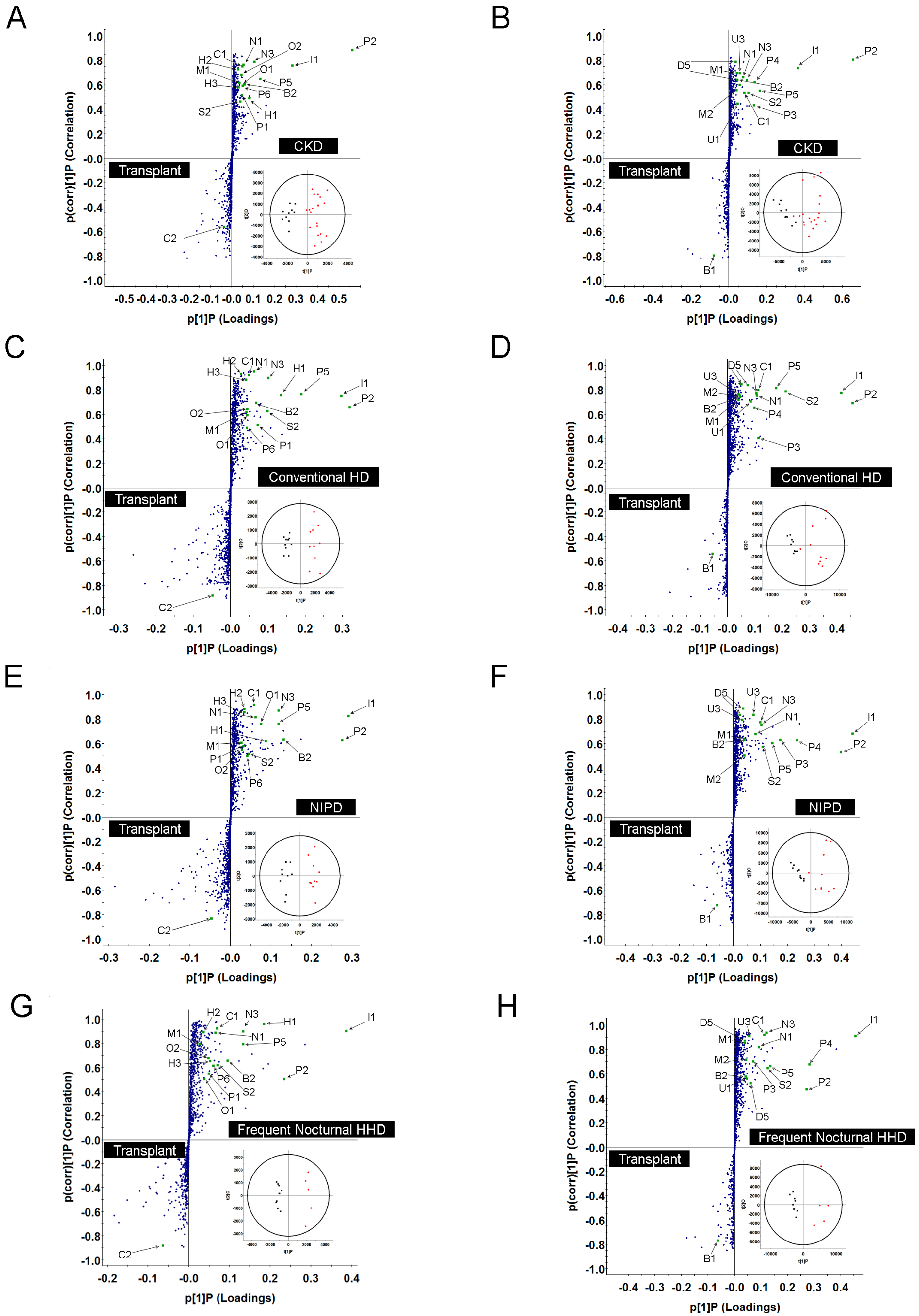

Supplemental Figure 3. Orthogonal partial least squares discriminant analysis (OPLS-DA) and S-plot projections comparing plasma metabolic features from kidney transplant (■, n = 10) patients with CKD (●, n = 20, A, RPLC; B, HILIC), conventional hemodialysis (conventional HD, ●, n = 10; C, RPLC; D, HILIC), nocturnal intermittent peritoneal dialysis (NIPD, ●, n = 10; E, RPLC; F, HILIC) and frequent nocturnal HHD (●, n = 5; HD E, RPLC; F, HILIC). Features are annotated (see Table 2 for metabolite list) with variable importance in projection (VIP) values > 1 and correlation (pcorr) values > 0.4 or < -0.4.

Supplemental Figure 4

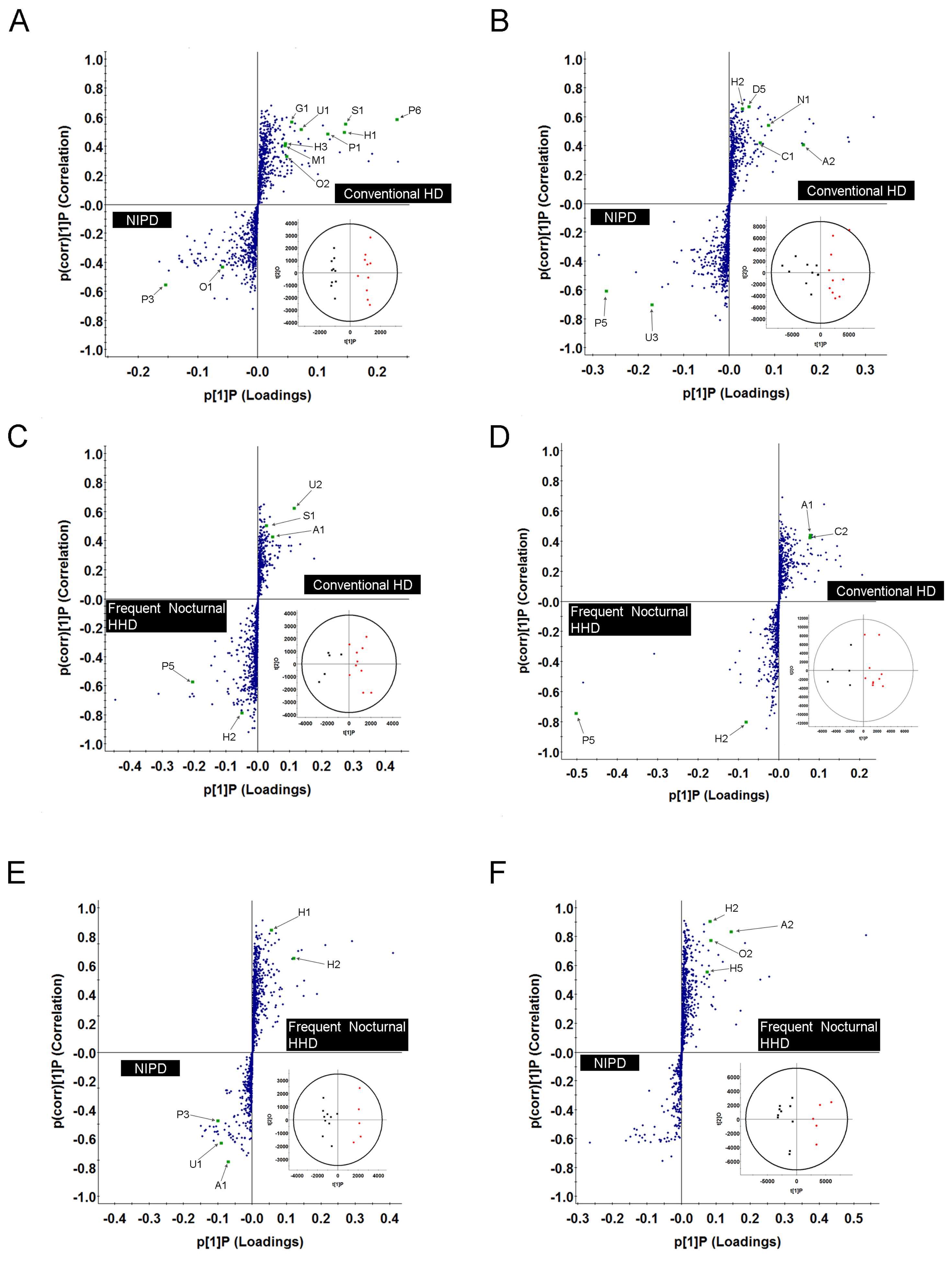

Supplemental Figure 4. Orthogonal partial least squares discriminant analysis (OPLS-DA) and S-plot projections comparing pre-dialysis plasma metabolic features between nocturnal intermittent peritoneal dialysis (NIPD, ■, n = 10) and conventional hemodialysis (conventional HD, ●, n = 10; A, RPLC; B, HILIC), frequent nocturnal HHD (■, n = 5) and conventional HD (●, C, RPLC; D, HILIC), and NIPD (■) and frequent nocturnal HHD (●, G, RPLC; H, HILIC). Features are annotated (see Table 2 for metabolite list) with variable importance in projection (VIP) values > 1 and correlation (pcorr) values > 0.4 or < -0.4.

Supplementary Figure 5

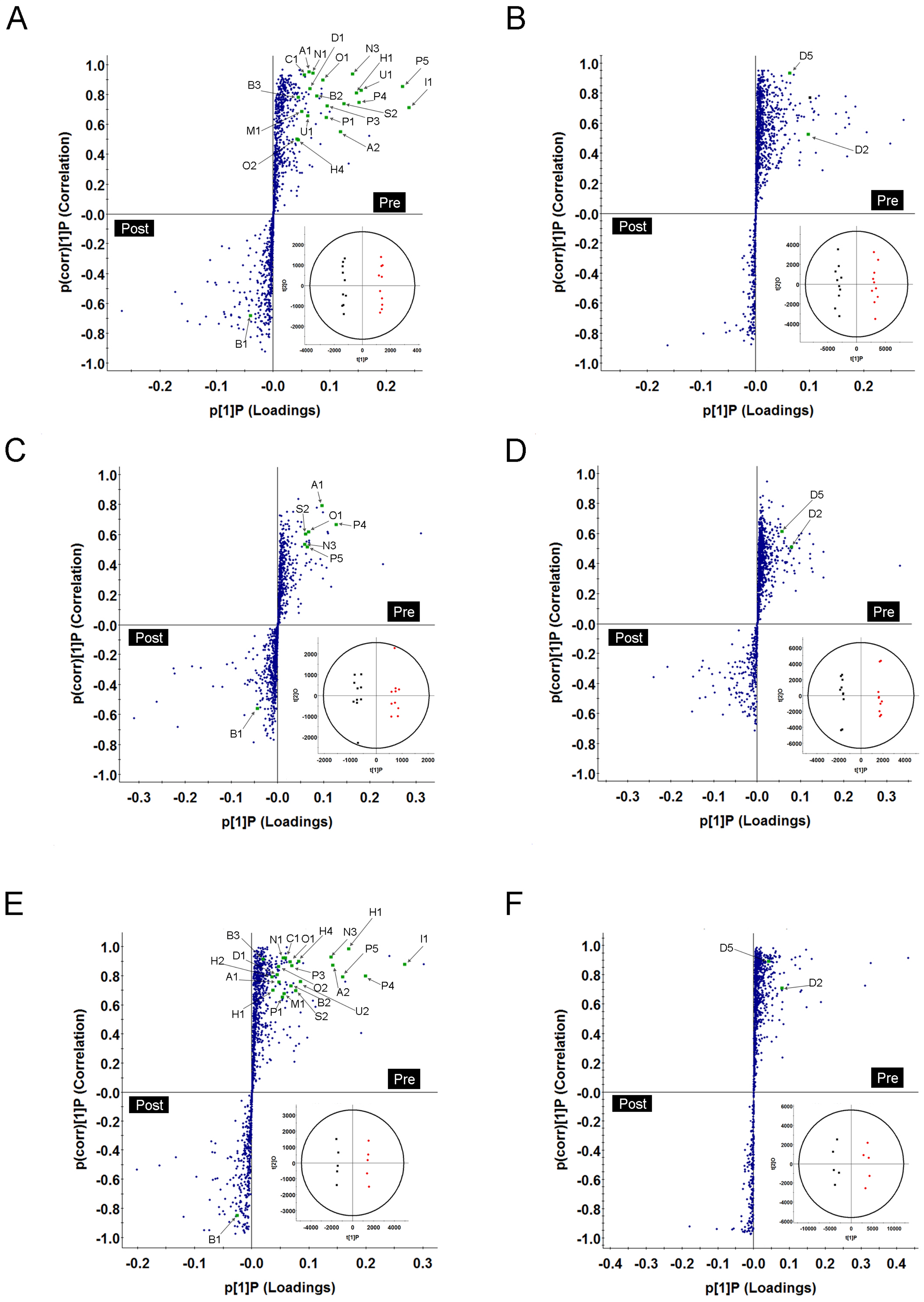

Supplemental Figure 5. Multilevel partial least squares discriminant analysis and S-plot projections of pre-(●) and post(■)-dialysis plasma samples from conventional hemodialysis (n = 10; A, RPLC; B, HILIC), nocturnal intermittent peritoneal dialysis (n = 10; C, RPLC; D, HILIC) and frequent nocturnal HHD (n = 5; E, RPLC; F, HILIC). Features are annotated (see Table 2 for metabolite list) with variable importance in projection (VIP) values > 1 and correlation (pcorr) values > 0.4 or < -0.4.

Supplemental Figure 6

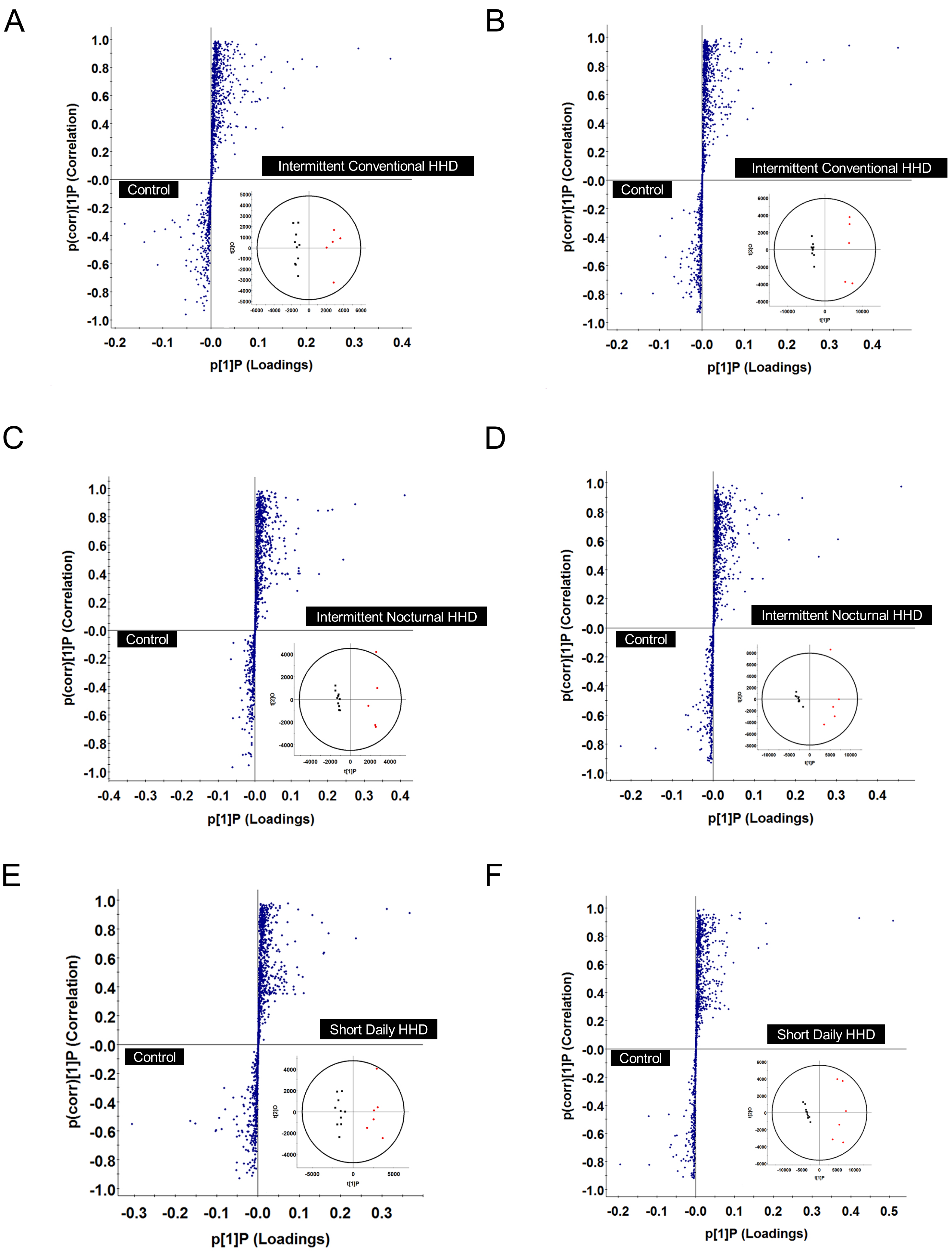

Supplemental Figure 6. Orthogonal partial least squares discriminant analysis (OPLS-DA) and S-plot projections comparing plasma metabolic features from control (■, n = 10) subjects with intermittent conventional HHD (●, n = 5; C, RPLC; D, HILIC), intermittent nocturnal HHD (●, n = 10; E, RPLC; F, HILIC) and short daily HHD (●, n = 6; HD E, RPLC; F, HILIC). Features are annotated (see Table 2 for metabolite list) with variable importance in projection (VIP) values > 1 and correlation (pcorr) values > 0.4 or < -0.4.

Supplemental Figure 7

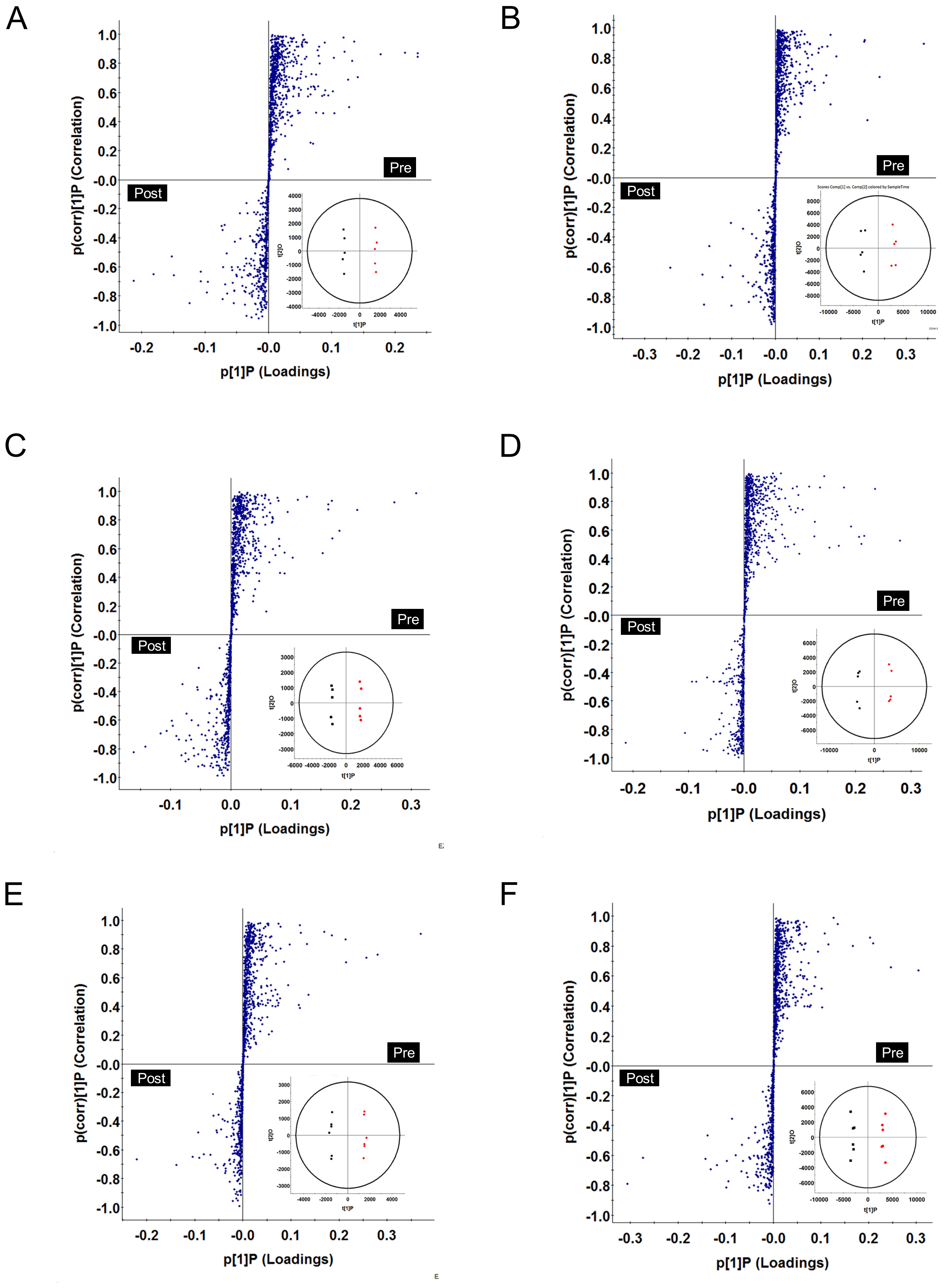

Supplemental Figure 7. Multilevel partial least squares discriminant analysis and S-plot projections of pre-(●) and post(■)-dialysis plasma samples from intermittent conventional HHD (n = 5; C, RPLC; D, HILIC), intermittent nocturnal HHD (n = 10; E, RPLC; F, HILIC) and short hours HHD (n = 6; HD E, RPLC; F, HILIC). Features are annotated (see Table 2 for metabolite list) with variable importance in projection (VIP) values > 1 and correlation (pcorr) values > 0.4 or < -0.4.
